# Efficacy of Soiled Bedding Transfer for Transmission of Mouse and Rat Infections to Sentinels: A Systematic Review

**DOI:** 10.1371/journal.pone.0158410

**Published:** 2016-08-12

**Authors:** W. C. C. de Bruin, E. M. E. van de Ven, C. R. Hooijmans

**Affiliations:** 1 QM Diagnostics BV, Nijmegen, The Netherlands; 2 Radboud University Medical Centre, SYRCLE at Central Animal Laboratory, Nijmegen, The Netherlands; Harvard University Faculty of Arts and Sciences, UNITED STATES

## Abstract

**Purpose:**

This systematic review was conducted to gain insight into the efficacy of transmission of infectious agents to colony sentinels by soiled bedding transfer based on publications studying this subject in mice and rats. This information is essential to establish recommendations for the design of health monitoring programs which use sentinels to determine the microbiological status of laboratory animal colonies.

**Results:**

Fifteen original articles retrieved from PubMed, Embase, and CAB abstracts met the inclusion criteria. The design of the studies varied substantially per infectious agent with regard to dose of soiled bedding, exposure time, and sentinel strains used.

**Conclusion:**

With our conservative criteria for effectiveness, soiled bedding transfer appeared to be effective for MHV, MPV, TMEV, *Helicobacter* spp., and fur mite infections and ineffective for Sendai virus. For other infectious agents, such as MNV, EDIM, MVM, SDAV, *Clostridium piliforme*, and pinworms, too few data were available to be able to draw robust conclusions on the efficacy of soiled bedding transfer.

**Recommendation:**

The identified evidence only pertains to a portion of the infectious organisms included in the FELASA 2014 guidelines. As many animal facilities design their health monitoring program according to these recommendations, additional studies are warranted to draw comprehensive conclusions on the effective transmission of the infectious agents listed in these guidelines by soiled bedding transfer.

## Introduction

Unwanted infections can affect the health of laboratory animals and humans (zoönoses), and also the reliability and interpretation of the results of scientific animal experiments. To ensure animal welfare, personnel welfare, and the reliability of the results from animal experiments, it is essential to know the microbiological status of the animals that are used in these studies. The Federation of Laboratory Animal Science Associations (FELASA) published guidelines to help animal facilities to develop health monitoring programs for rodents and rabbits [1] [2]. These guidelines contain recommendations not only for the infectious agents that should be tested, but also for the use of sentinels, testing of biological samples, which infectious agents should be tested, frequency of testing, and sample size.

To detect viral, bacterial, and parasitic infections, in rodents, many facilities use sentinels that are exposed to soiled bedding of other animals housed in the same microbiological unit. It is assumed that the outcome of sentinel health monitoring reflects the microbiological status of the entire colony. Currently, there is renewed interest in the efficacy of microbiological monitoring using soiled bedding sentinels as a growing number of laboratory animals are housed in individually ventilated cage (IVC) rack systems. Each IVC cage is considered to be a microbiological unit, and the IVC has been designed to prevent cage-to-cage transmission of infections. Transfer of animals between IVC cages is performed in changing stations to mitigate changes in the microbiological status of the cages.

For ethical reasons, such as animal welfare and responsible laboratory animal use, it is important to know whether the use of soiled bedding sentinels in health monitoring can be justified. A preliminary search of the published literature showed that protocols and standards for bedding transfer vary widely. These variations lead to a number of open questions: What infectious agents can be transmitted to sentinel animals by soiled bedding transfer? Is transmission of primarily airborne infectious agents possible using sentinels in IVC systems? Does the efficacy of the soiled bedding transfer depend on the sentinel strain that is used? How much soiled bedding should be transferred and how frequently for transmission of infectious agents to be effective? For what length of time should sentinels be exposed to dirty bedding to become infected?

In order to undertake to answer the above mentioned questions and to establish recommendations for the design of an effective sentinel health monitoring program, we systematically reviewed the available evidence concerning the efficacy of transmission of unwanted infectious organisms to sentinels using soiled bedding.

## Materials and Methods

### Search strategy and selection of papers

This systematic review was performed to determine the efficacy of transmission of infection to sentinels using soiled bedding transfer. Papers were included if they studied the efficacy of transmission of infections with mouse and rat infectious agents included in the FELASA guidelines using soiled bedding transfer. Studies were excluded if no control group, e.g., animals receiving clean bedding or bedding from non-infected animals, was present in the experimental design or if the control group became infected during the experiments. Inclusion and exclusion criteria were defined and documented in a protocol before the start of the study.

**Fig 1:**
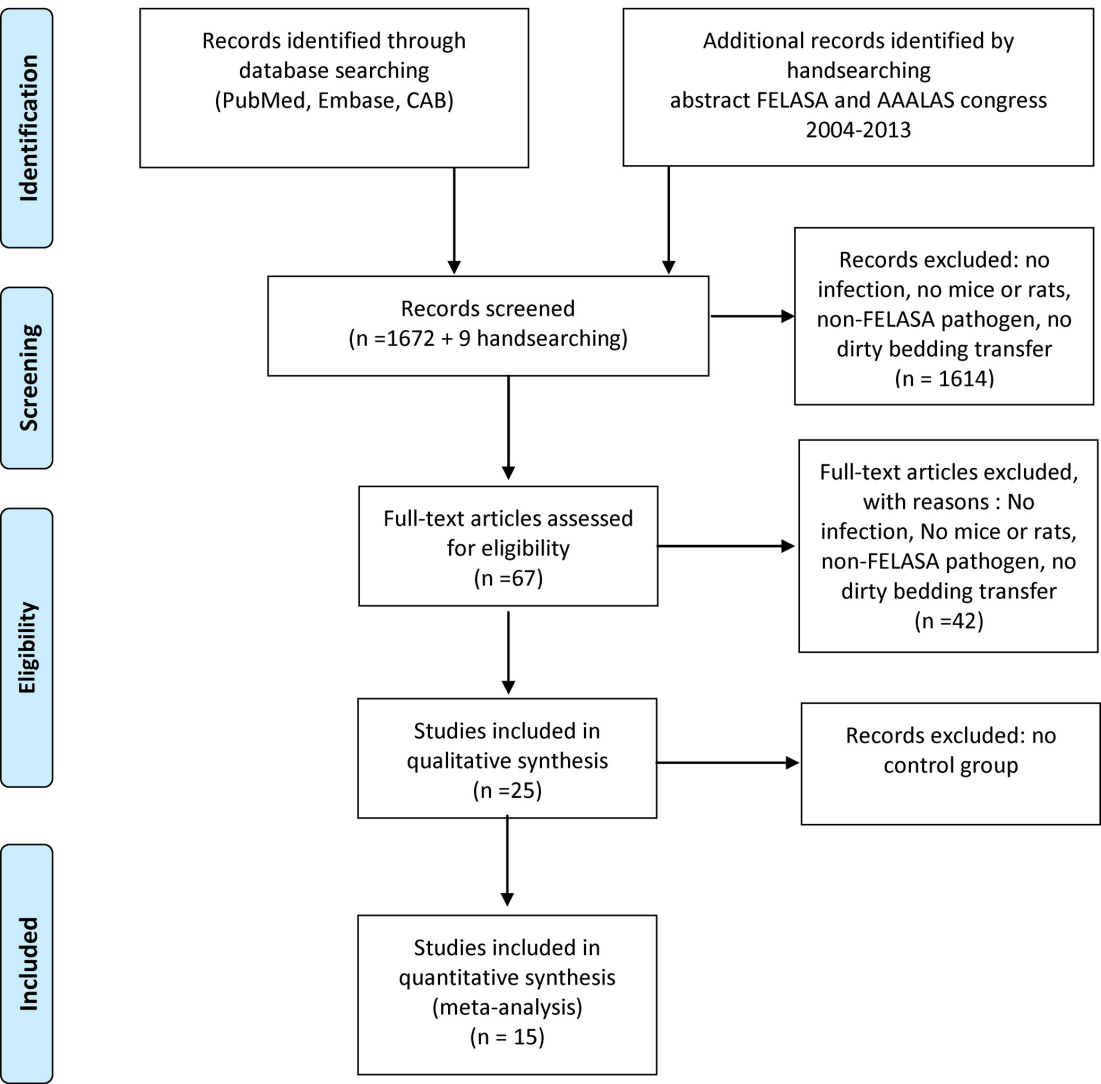
PRISMA 2009 Flow Diagram of the search strategy.

PubMed, Embase, and CAB abstracts were searched for all original articles until October 2014. In addition, the abstract books of the FELASA (2004, 2007, 2010, 2013) and AALAS congresses (2003–2013) were hand-searched. The search strategy, as depicted in Fig 1, included the following four components: infection, sentinel, bedding transfer, and mouse and/or rat. To detect all mouse and rat studies in PubMed, Embase, and CAB abstracts as described by [3], the search filters were set to include rat and mouse studies only. Duplicate studies were removed.

Study selection was performed independently by W. de Bruin (WB) and E. van de Ven (EV). Differences were resolved by the third author (C. Hooijmans).

No language restrictions were applied.

### Study characteristics and data extraction

As shown in S2 File, the following characteristics were extracted from the included studies: reference list number, animal species, sentinel strain, sentinel age in weeks at start of bedding transfer, sex, immune-competence of the sentinel strain, number of animals per group, number of sentinels per cage, type of control group, proven infection in source animals, infectious agent studied, time of bedding exposure, change of bedding in times per week, doses of soiled bedding, type of housing, type of diagnostic test applied to prove infection, outcome measure of the experimental group and outcome measure of the control group. Papers were only included if no infection in the control groups was demonstrated. When data could not be retrieved from the paper, the authors were contacted to obtain this information.

When more than one paper was included on the transmission of infection with a certain infectious agent by soiled bedding, the outcome measures for each individual paper were identified and these were compared with each other. This comparison then led to an overall conclusion on the efficacy of transmission of infection with a particular infectious agent by soiled bedding transfer.

### Assessment of methodological and reporting quality

The quality of the included studies and their risk of bias were independently assessed by two reviewers (WB, EV). In order to study risk of bias of the included papers, an adapted version of the SYRCLE risk of bias tool was used [3] [4]. The risk of selective outcome reporting and the risk of attrition bias were not assessed as it is still uncommon for animal studies to pre-specify and publish details of planned experiments. This paper focused on selection bias, performance bias, and detection bias.

As described in Table 1, the following items, were assessed for randomization: sequence generation, random housing, and random outcome assessment. Three items were assessed for blinding: allocation concealment, blinding of trial caregivers (animal caretakers) and researchers, and blinding of outcome assessors. A “yes” judgment (green) indicates low risk of bias; a “no” judgment (red) indicates high risk of bias; an “?” judgment (orange) indicates that insufficient details were reported to be able to properly assess risk of bias.

**Table 1:** Results of quality assessment, randomization and blinding.

Ref.	Rando- mization	Blinding	Baseline charac-teristics similar^#^	Sequence generation	Random housing	Random outcome assessment	Allocation concealment	Blinding trial caregivers and researchers	Blinding outcome assessors
[7]	R	NR	L						
[8]	R	R*							
[9]	NR	NR	L						
[10]	NR	R	L						
[11]	NR	NR	L						
[12]	NR	R*	L						
[13]	NR	NR							
[14]	NR	R	L						L, ^1^
[15]	NR	NR	L						
[16]	NR	NR							
[17]	NR	R*							
[18]	R	NR							
[19]	NR	R*	L						
[20]	NR	R*							
[21]	NR	NR	L						

NR = not reported, R = reported.

^#^ = baseline characteristics are checked for age, sex, strain and housing conditions, Ly = low risk of bias, white = unclear risk of bias,. R* = it was assumed that the diagnostic samples were blinded as samples had been submitted to a commercial tester.

^**1**^ = all samples were read by two independent readers blinded for the intervention. The samples were marked by persons other than those who read the slides.

As reporting of essential details in animal studies is often poor [5]. two reporting quality indicators, randomization and blinding, were also inserted. Per reference, it was assessed whether experiments were randomized or blinded at any level.

### Data synthesis

In order to be able to draw conclusions on the efficacy of transmission of infections to sentinels by soiled bedding, the results of the analysis had to fulfill the following three criteria:

1. At least one of the exposed sentinels needed to be infected with the infectious agent.
2. The outcome measure of two independent studies on the efficacy of bedding transfer should be 100% to be considered effective and was scored as partially effective at 50% consistency.
3. The outcome measure of more than two independent studies on the efficacy of transmission of infection by soiled bedding transfer was scored as follows: 0–25% consistency as poorly effective (no), 25%-75% as partially effective (partial), and 75%-100% as effective (yes).

With regard to the second criterion, we underline that the presence of a single study or two independent studies with inconsistent results does not mean that the results are not reliable, but that there is not enough evidence to be able to draw conclusions and that further research into that specific infectious agent is necessary.

## Results

### Study selection and characteristics

As shown in the PRISMA 2009 Flow Diagram [6] in Fig 1, the electronic search strategy as displayed in the S1 File, retrieved 1890 papers. Nine additional references were obtained from screening the abstract books of the 2003–2013 FELASA and the American Association for Laboratory Animal Science AALAS congresses. After excluding duplicates, a total of 1681 abstracts were screened. We excluded abstracts that did not describe transmission of infections with infectious agents included in the FELASA 2014 recommendations with mice or rats via dirty bedding transfer. A total of 67 papers were selected for full-text screening, of which 25 original papers remained using the inclusion and exclusion criteria [7–21]. Finally, ten articles were excluded, because no control group had been used in the experiments, resulting in a total of fifteen papers for qualitative synthesis.

The characteristics of the fifteen included studies have been summarized in S2 File. There was great variability in the design of the studies. IVCs were used to house the animals in eleven studies, filter top cages (FTC) in three studies, and semirigid isolators (SRIs) in one study. Of the fifteen included studies, two were performed with rats, twelve with mice, and one study used both mice and rats. All rat studies (n = 3) used female Spraque Dawley (SD) rats, but each studied a different infectious agent (i.e., *Clostridium piliforme* (CP), CAR bacillus and sialodacryoadenitis virus (SDAV).

In the other thirteen studies, a total of nineteen different mouse strains or stocks were used: CR:ORL Sencar (n = 1); Tac Swiss Webster (n = 2), CD-1 strains (n = 6), C57BL/6NCr (n = 1), Balb/cAnNHsd (n = 1), SCID (n = 1), B6C3F1 (n = 1), C3H/HeN (n = 1), VAF Mice (n = 2), MTV (n = 1), RMTV (n = 1) and DBA/2N (n = 1). These studies examined the transmission of infection with murine norovirus (MNV), mouse hepatitis virus (MHV), mouse parvovirus (MPV), minute virus of mice (MVM), Sendai virus (Sendai), murine rotavirus (EDIM), Theiler's mouse encephalomyelitis virus (TMEV), mouse adenovirus (MadV), fur mites, pinworms, endoparsites other than pinworms, *Pasteurella pneumotropica*, *Pseudomonas aeruginosa*, *Helicobacter* spp., and *Pneumocystis murina*. In one study, transmission of SDAV was studied in mice, although the mouse is to our knowledge not susceptible to this infectious agent, and SDAV will not cause infections in mice. Nine of the twelve studies used only female mice, one only male mice, one used both sexes, and sex was not reported in two studies.

Before the start of the experiments, the sentinel animals used in all studies had been proven negative for the investigated pathogens according to the annual test profile of the FELASA guideline for health monitoring of mice and rats. All source mice whose soiled bedding was used for transfer to the sentinel cages had been proven positive for the investigated infectious agent before soiled bedding transfer took place. The doses of bedding transferred to the sentinel cages varied among the studies from a spoon to a cupful, or 2.5%, to 100% soiled bedding sampled from 1 to 84 source cages.

Depending on the infectious agent, different diagnostic tools were used to determine the outcome measurements. For viral pathogens, CAR bacillus, and Clostridium piliforme, serological techniques such as enzyme-linked immunosorbent assay (ELISA), immunofluorescent assay (IFA), and multiplex fluorescent immunoassay (MIA) were applied to determine the presence of antibodies against the infectious agent as proof of active infection. Detection of Helicobacter species and Pneumocystis murina was performed using polymerase chain reaction (PCR). MHV and MPV were tested for both by serology and PCR. Pasteurella pneumotropica was tested for both by bacterial culture and PCR. Pseudomonas aeruginosa was tested for by bacterial culture. Parasites were demonstrated by both microscopy and and PCR.

### Risk of bias and quality of reporting

The results of the quality assessment of the fifteen included studies are shown in Table 1. Randomization and blinding was poorly reported in the studies included in this systematic review: only 13% (2/15) of the included studies reported randomization or blinding at any level of the experiment. In 30% (5/15) of the studies, we assumed that the diagnostic samples were blinded, because samples had been submitted to a commercial tester.

Baseline characteristics appeared to be comparable in 60% (9/15) of the studies. In the other six papers, the baseline characteristics of the included animals (e.g., age, sex, and strains) were not all clearly described. As a consequence of poor reporting of essential methodological details in the included papers, an unclear risk of bias was scored for all other risk of bias items.

### Data synthesis: efficacy of soiled bedding transfer

Transmission of pathogens to sentinel animals by soiled bedding was considered to be effective if at least one of the exposed sentinels became infected. Based on this first criterion, soiled bedding transfer appears to be successful in mice for MNV, MHV, TMEV, MVM, MPV, *Helicobacter* spp., *Pasteurella pneumotropica*, *Pseudomonas aeruginosa*, *Pneumocystis murina*, fur mites, pinworms, and endoparasites other than pinworms (Table 2). In rats, soiled bedding transfer appears to be successful for SDAV and *Clostridium piliforme*.

**Table 2:** Efficacy of transfer of pathogens via soiled bedding and accordance of results between the included studies.

Pathogen	Author	Conclusion: Bedding transfer effective per experiment^1)^	Meta conclusion:Effective bedding transfer ^2)^
**Mouse**			
EDIM	Compton, 2004 [10]	no	n.a.
Endoparasites other than pinworm	Brielmeijer, 2006 [8]	yes	n.a.
*Helicobacter* spp.	Compton, 2004 [10]	yes	yes (100%)
	Henderson, 2013 [20]	yes	
	Livingston, 1998 [15]	yes	
	Myers, 2003 [18]	yes	
Fur mites	Arbona, 2010 [7]	yes	yes (100%)
	Henderson, 2013 [20]	yes	
	Lindstrom, 2011 [14]	yes	
	Thigpen, 1989 [19]	yes	
MAdV	Henderson, 2013 [20]	no	n.a.
MPV	Brielmeijer, 2006 [8]	no	yes (75%)
	Compton, 2004 [10]	yes	
	Compton, 2012 [21]	yes	
	Henderson, 2013 [20]	yes	
MNV	Manuel, 2008 [16]	yes	n.a.
MVM	Henderson, 2013 [20]	yes	n.a.
*Pasteurella pneumotropica*	Henderson, 2013 [20]	no	n.a.
	Myers, 2003 [18]	yes	
Pinworms	Henderson, 2013 [20]	yes	n.a.
*Pneumocystis murina*	Myers, 2003 [18]	yes	n.a.
*Pseudomonas aeruginosa*	Henderson, 2013 [20]	yes	n.a.
Sendai	Compton, 2004 [10]	no	no (100%)
	Dillehay, 1990 [12]	no	
SDAV	La Regina, 1992 [13]	no	n.a.
TMEV	Brownstein, 1981 [9]	yes	yes (100%)
	Henderson, 2013 [20]	yes	
**Rat**			
CAR bacillus	Cundiff, 1995 [11]	no	n.a.
*Clostridium piliforme*	Motzel, 1992 [17]	yes	n.a.
SDAV	La Regina, 1992 [13]	yes	n.a.

^1)^ inclusion criterion: yes = at least one of the sentinels is infected; no = none of the sentinels is infected

^2)^Meta conclusion on efficacy of bedding transfer based on consistent outcome in the studies included in the comparison. Yes = effective, no = ineffective transfer of pathogens via soiled bedding transfer. N.a. = not applicable in case only one study is included or two studies with inconsistent results are included.

We could only draw conclusions on transmission efficacy on the basis of consistency of results so limited us to seven pathogens for which more than one study had been included in this systematic review (Table 2). When we combined this second criterion with the pathogens selected with the first criterion, sufficient data to conclude that these agents are effectively transmitted via soiled bedding transfer were only available for MHV, MPV, TMEV, *Helicobacter* spp., and fur mites.

Effective transmission of *Clostridium piliforme*, MNV, MVM, pinworms, *Pneumocystis murina*, SDAV, and endoparasites other than pinworms were only described in one paper. As a consequence we could draw no robust conclusions on the efficacy of transmission of these infections via soiled bedding transfer. The two papers on transmission of *Pasteurella pneumotropica* showed conflicting results. As a result, we conclude that transmission of this infection via soiled bedding transfer is only partially effective.

Transmission of infection by soiled bedding transfer was not established for Sendai virus, EDIM, MAdV, SDAV, and *Pseudomonas aeruginosa* in mice and CAR bacillus in rats. For Sendai virus, the two included papers consistently describe that this infectious agent is not effectively transferred by soiled bedding transfer. Because of this low number of studies no robust conclusions on the efficacy of transmission of these infections via soiled bedding transfer could be drawn for EDIM, MAdV, SDAV, *Pseudomonas aeruginosa*, and CAR bacillus.

## Discussion

It is hotly debated among laboratory animal veterinarians and colony managers if soiled bedding sentinels are an effective method of determining the health status of animals housed in IVC systems. Alternative methods proposed are: testing biological samples received from colony animals randomly selected from the rack by live animal sampling, or testing samples taken from the IVC rack (e.g., swab of the plenum or a sample of the rack filters) [20,22].

Testing samples received from randomly selected animals or from the IVC rack appears to be preferred as this involves a reduction in the number of animals used for health monitoring and no transport of animals to the diagnostic laboratory is required. Bedding transfer, moreover, is labor-intensive, and the quality of bedding transfer by animal caretakers cannot easily be controlled in everyday practice.

The efficacy of transmission of mouse and rat pathogens to sentinel animals by soiled bedding transfer should be made evident. Only then can an accurate comparison be made between the use of soiled bedding transfer, biological samples received via live animal sampling of randomly selected animals from the rack, or testing air exhaust dust samples of rack plenum and filters for health monitoring purposes. This is mandatory to enable laboratory animal facility managers to make an informed decision about the best method to provide reliable health monitoring in IVCs.

In this paper, we show that the literature on soiled bedding transfer is scarce and that there are considerable variations in design between the few studies that have been conducted with regard to dose of soiled bedding, exposure time, number of animals in experimental group, and sentinel strains used. However, based on our conservative criteria for effectiveness, soiled bedding transfer appeared to be effective to detect MHV, MPV, TMEV, *Helicobacter* spp., and fur mite infections in the source colony and ineffective for Sendai virus. As a consequence of the few available studies and the large variation in study designs, no comprehensive recommendations for the design of an effective sentinel health monitoring program could be proposed.

For *Pasteurella pneumotropica*, bedding transfer appears to be partially effective. For other infectious agents, such as C*lostridium piliforme*, *Pseudomonas aeruginosa*, MNV, MVM, SDAV, and pinworms, too few data were available (according to our criteria for effectiveness) to be able to draw robust conclusions. This is also counts for EDIM, Endoparasites other than pinworms, and MAdV for which only one paper was included, in which soiled bedding transfer was reported not to be effective. The fact that not enough evidence is available to draw robust conclusions regarding those infections, does not mean that those studies in themselves were not valuable.

MHV, MPV, TMEV, *Helicobacter* spp. for which soiled bedding transfer appeared to be effective, are all spread through the fecal oral route [9, 8,14]. C*lostridium piliforme*, *Pseudomonas aeruginosa*, MNV, MVM, SDAV, EDIM, endoparasites, and MAdV are transmitted in the same way [12, 15, 16, 20]. This also makes them good candidates for effective transmission via soiled bedding transfer. The included papers for MNV, MVM endoparasites, C*lostridium piliforme and Pseudomonas aeruginosa* seem to confirm this assumption, whereas the papers for EDIM and MAdV show the opposite.

Sendai virus is a respiratory agent and is not effectively transmitted through soiled bedding transfer. The papers included for CAR bacillus, *Pneumocystis murina and Pasteurella pneumotropica* do not provide sufficient evidence to draw a conclusion that respiratory agents can’t be transmitted through soiled bedding transfer. Therefore additional studies are urgently needed to determine efficacy of the transmission of these infectious agents through soiled bedding transfer.

The study details of the included studies have been very poorly reported, which is worrying as non-reporting of important methodological details might be indicative of the neglected use of these methods to reduce bias, causing skewed results [23]. As a consequence of poor reporting of essential methodological details, the risk of bias level could not be assessed in most studies, and no meta-analysis could be performed. This seriously hampers our attempt to draw reliable conclusions from the animal studies included in this systematic review.

Soiled bedding transfer to transmit infection to sentinels is a commonly accepted method for health monitoring of rodent colonies used in most animal facilities worldwide. This systematic review shows that, on the basis of our criteria, there is only enough evidence to substantiate that MHV, MPV, TMEV, *Helicobacter* spp., and fur mites are effectively transmitted via soiled bedding transfer. Even for these pathogens, however, it is not evident what the minimal demands are for effective soiled bedding transfer: What dose of soiled bedding should be used, which sentinel strains should be used, and what should the frequency of bedding transfer be? The results described in Lindstrom et al. [14], as shown in Table 1, clearly demonstrate that dose of bedding has an impact on the efficacy of bedding transfer as fur mites were only transmitted to the sentinel at the highest dose of bedding transferred.

Additional studies, therefore, are warranted to be able to draw conclusions on the efficacy of soiled bedding transfer for every infectious agent listed in the FELASA guidelines. The outcomes will hopefully enable the design of a good health monitoring program using soiled bedding sentinels. A health monitoring program of pathogens for which soiled bedding transfer has proven to be effective should outline preferred sentinel strain, dose of bedding to be transferred, and frequency and exposure time of soiled bedding transfer. These future experiments should adhere to the Gold Standard Publication Checklist (GSPC) [24] and Animal Research: Reporting In Vivo Experiments (ARRIVE) [25] guidelines in order to produce more robust and more reliable results.

## Supporting Information

S1 File Search strategy SRV. (DOCX)

S2 File Characteristics of the included studies. (XLSX)
