# Supplementary material for: Efficacy of Soiled Bedding Transfer for Transmission of Mouse and Rat Infections to Sentinels: A Systematic Review
Source: PLoS One. 2016 Aug 12;11(8):e0158410. doi: 10.1371/journal.pone.0158410 (PMC4982683; doi:10.1371/journal.pone.0158410)
Supplement: S1 File — (DOCX) [file pone.0158410.s001.docx]

**Search strategy SR**

(("Soiled bedding"[tiab] OR (soiled[tiab] AND ("bedding and linens"[MeSH Terms] OR ("bedding"[tiab] AND "linens"[tiab]) OR "bedding and linens"[tiab] OR "bedding"[tiab])) OR "Contaminated bedding"[tiab] OR (contaminated[tiab] AND ("bedding and linens"[MeSH Terms] OR ("bedding"[tiab] AND "linens"[tiab]) OR "bedding and linens"[tiab] OR "bedding"[tiab])) OR "Dirty bedding"[tiab] OR (dirty[tiab] AND ("bedding and linens"[MeSH Terms] OR ("bedding"[tiab] AND "linens"[tiab]) OR "bedding and linens"[tiab] OR "bedding"[tiab])) OR "Bedding transfer"[tiab] OR "Fecal-oral"[tiab] OR "sentinel program"[tiab] OR "quality assurance testing"[tiab] OR "quality assurance system"[tiab] OR "quality assurance systems"[tiab] OR "health monitoring"[tiab] OR "health control"[tiab] OR "animal health surveillance"[tiab] OR "microbiological monitoring"[tiab] OR seromonitoring[tiab] OR Bacteriological Monitoring[tiab] OR Bacteriologic Monitoring[tiab] OR Virologic Monitoring[tiab] OR Virological Monitoring[tiab] OR Parasitological Monitoring[tiab] OR Serological Monitoring[tiab] OR Serologic Monitoring[tiab] OR Bacteriological Surveillance[tiab] OR Bacteriologic Surveillance[tiab] OR Virologic Surveillance[tiab] OR Virological Surveillance[tiab] OR Parasitological Surveillance[tiab] OR Parasitologic Surveillance[tiab] OR Serological Surveillance[tiab] OR Serologic Surveillance[tiab] OR Bacteriological Control[tiab] OR Bacteriologic Control[tiab] OR Virologic Control[tiab] OR Virological Control[tiab] OR Parasitological Control[tiab] OR Serological Control[tiab] OR Serologic Control[tiab]) AND ("Mice"[Mesh] OR "Rats"[Mesh] OR "rodentia"[MeSH Terms] OR mice[Tiab] OR mus[Tiab] OR mouse[Tiab] OR murine[Tiab] OR woodmouse[tiab] OR rats[Tiab] OR rat[Tiab] OR murinae[Tiab] OR muridae[Tiab] OR cottonrat[tiab] OR cottonrats[tiab] OR rodentia[Tiab] OR rodent[Tiab] OR rodents[Tiab] OR sentinel[tiab] OR sentinels[tiab] OR wistar[tiab] OR C57bl/6[tiab] OR Balb/c[tiab] OR spraque dawley[tiab] OR F344[tiab])) OR ((((("Rodent Diseases/microbiology"[Mesh] OR "Rodent Diseases/parasitology"[Mesh] OR "Rodent Diseases/transmission"[Mesh] OR "Rodent Diseases/virology"[Mesh]) OR ("Disease Transmission, Infectious/veterinary"[Mesh]) OR ("Mice/microbiology"[Mesh] OR "Mice/parasitology"[Mesh] OR "Mice/virology"[Mesh]) OR ("Rats/microbiology"[Mesh] OR "Rats/parasitology"[Mesh] OR "Rats/virology"[Mesh]))) AND ((("Sentinel Surveillance"[Mesh])) OR ("Sentinel Surveillance/veterinary"[Mesh]))) OR ((((("Sentinel Surveillance"[Mesh])) OR ("Sentinel Surveillance/veterinary"[Mesh])) OR ("Disease Transmission, Infectious/veterinary"[Mesh]) OR ("Rodent Diseases/microbiology"[Mesh] OR "Rodent Diseases/parasitology"[Mesh] OR "Rodent Diseases/transmission"[Mesh] OR "Rodent Diseases/virology"[Mesh]) OR ("Mice/microbiology"[Mesh] OR "Mice/parasitology"[Mesh] OR "Mice/virology"[Mesh]) OR ("Rats/microbiology"[Mesh] OR "Rats/parasitology"[Mesh] OR "Rats/virology"[Mesh])) AND (("Animal Welfare"[Mesh]) OR ("Housing, Animal"[Mesh]))))
